# Successful Treatment of Cutaneous Mycobacterium chelonae Infection in an Immunocompromised Patient Using Moxifloxacin and Clarithromycin

**DOI:** 10.7759/cureus.76628

**Published:** 2024-12-30

**Authors:** Hanako Miyahara, Enmi Yamada, Sawako Kiyohara, Shijima Taguchi, Mikiro Kato

**Affiliations:** 1 Department of Dermatology, Mito Kyodo General Hospital, University of Tsukuba, Mito, JPN; 2 Department of Infectious Diseases, University of Tsukuba Hospital, Tsukuba, JPN

**Keywords:** antibiotic, biopsy, immune compromisation, mycobacterium chelonae, nontuberculous mycobacterial infection

## Abstract

*Mycobacterium chelonae *is a ubiquitous organism classified as a nontuberculous mycobacterium that rarely causes indolent skin or soft-tissue infections, especially in immunocompromised patients. Given the rarity of *M. chelonae *infection, diagnosis can be difficult because cutaneous lesions may be considered a worsening of the underlying disease or a benign condition. Here, we report a case of a rapidly progressing cutaneous *M. chelonae *infection in a patient with nephrotic syndrome. The cutaneous lesion was initially stable for five months, mimicking noninfectious etiologies; however, due to rapid worsening, prompt skin biopsy revealed *M. chelonae *infection. Clinicians should be aware of this unusual presentation of *M. chelonae *infection and have a low threshold for performing biopsies for the early diagnosis and prevention of disease progression. The patient was successfully treated using a combination of moxifloxacin and clarithromycin. The choices of antibiotics and duration of treatment for nontuberculous mycobacterial infections are not clearly determined and depend on the clinician's experience. As treatment duration is prolonged, it sometimes carries the risk of developing drug resistance. We also evaluated the efficacy of moxifloxacin and clarithromycin for treating cutaneous *M. chelonae *infections in immunocompromised patients who require long-term antibiotic therapy.

## Introduction

The incidence of nontuberculous mycobacterial (NTM) infections has increased in immunocompromised patients [1]. NTM species are classified into four groups according to the Runyon system based on their growth rate and pigment production. *Mycobacterium chelonae* belongs to group IV and grows faster than other NTM species [1]. *M. chelonae* is a ubiquitous organism that grows at 25-33 °C, with a preference for temperatures of 30-32 °C [1,2]. Because of its ability to grow at lower temperatures, *M. chelonae* infections typically present as cutaneous infections of the extremities [3]. Immunocompetent patients develop localized cutaneous infections after trauma, surgery, intravenous catheter placement, or tattooing with contaminated instruments [4]. Conversely, immunosuppressed patients typically present with chronic syndrome with multiple slowly progressing nodules [5]. A definitive diagnosis is made after a skin biopsy and culture study; however, as the timing of the biopsy depends on the clinical scenario, the diagnosis may be delayed if clinicians do not consider *M. chelonae* infection as a differential diagnosis. No published treatment guidelines and no randomized controlled trials comparing different therapeutic regimens exist for *M. chelonae* infections [4]; therefore, ideal antibiotic regimens based on disease severity and host immune status have not yet been determined. Herein, we present a unique case of cutaneous *M. chelonae* infection in an immunocompromised patient. Although the single nodule was stable for five months, mimicking noninfectious etiologies, it progressed rapidly within a few days. We emphasize the importance of performing an immediate biopsy in immunocompromised patients, even for stable single nodules, when considering *M. chelonae* infection as a differential diagnosis. We also show the effectiveness of the combination of moxifloxacin and clarithromycin for treating progressive cutaneous *M. chelonae* infection in immunocompromised patients.

## Case presentation

A 68-year-old male with nephrotic syndrome presented to our hospital with a painless nodule on his left lower leg that had been present for five months since August 2022. Comorbidities included ischemic heart disease and hyperlipidemia. The patient had received high-dose steroid therapy for relapsing nephrotic syndrome two months before his presentation: intravenous methylprednisolone 500 mg daily for three days followed by 40 mg oral prednisolone daily for six weeks, then tapered by 5 mg every two to four weeks. This led to an extended hospital stay for treating Pneumocystis pneumonia infection. The patient was discharged with a prescription of 35 mg oral prednisolone and 75 mg cyclosporine daily one week before his presentation to our hospital.

**Figure 1:**
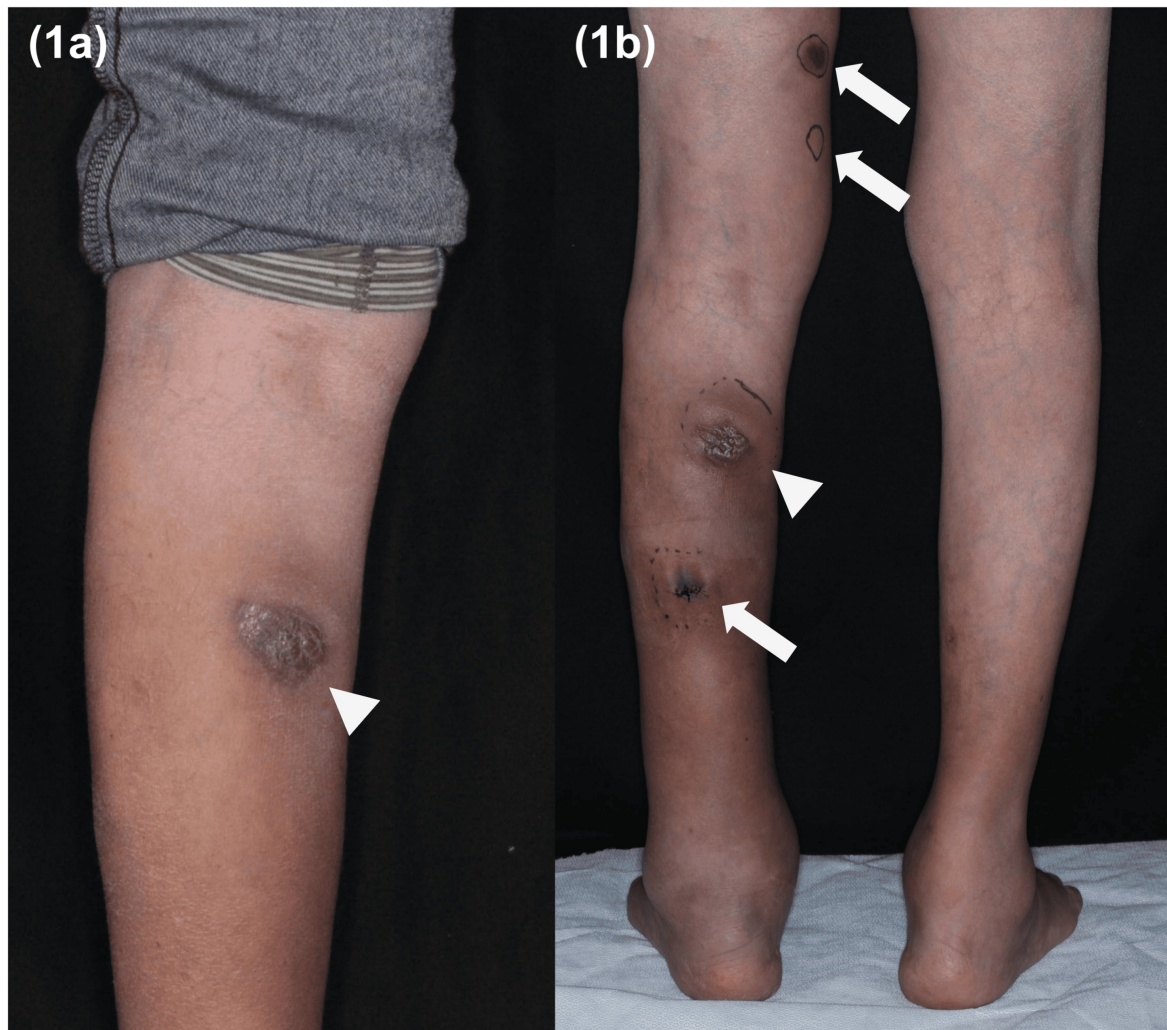
Clinical images. (a) Well-defined nodules with subcutaneous induration on the posterior aspects of the left lower leg at initial visit (*arrowhead*). (b) Multiple nodules with subcutaneous induration on the posterior of the left leg two weeks later (*arrows*).

Physical examination revealed a well-defined 5 × 2 cm painless solid nodule with subcutaneous induration on the posterior side of his left lower leg (Figure 1a). The patient did not have systemic symptoms such as fever, loss of weight, or loss of appetite. The initial differential diagnosis was either scarring from insect bites or squamous cell carcinoma. The patient was treated with a topical betamethasone butyrate propionate ointment 0.05%, and a skin biopsy was planned in case of no improvement. Two weeks later, the nodule had enlarged and spread to the left lower leg along the lymphatic system as multiple painful nodules (Figure 1b).

**Figure 2:**
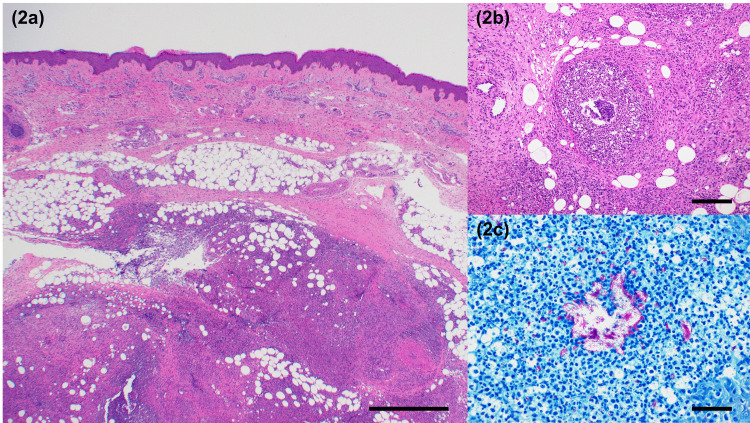
Histopathology of the primary nodule of the left lower leg. (a, b) Hematoxylin/eosin stain. Neutrophilic infiltration and granuloma with vacuoles from the dermis to the subcutaneous fat tissue. (c) Ziehl-Neelsen stain. Acid fast bacilli were observed in vacuoles. a, bar = 1 mm; b, bar = 200 μm; c, bar = 50 μm.

A skin biopsy was performed, and the specimen was sent for pathological and culture studies. Histopathological examination revealed a granuloma with vacuoles and neutrophilic infiltration from the dermis to the deep subcutaneous fat tissue (Figure 2a, b), where they were the most dominant. Numerous bacteria were observed in these vacuoles on Ziehl-Neelsen staining (Figure 2c).

**Figure 3:**
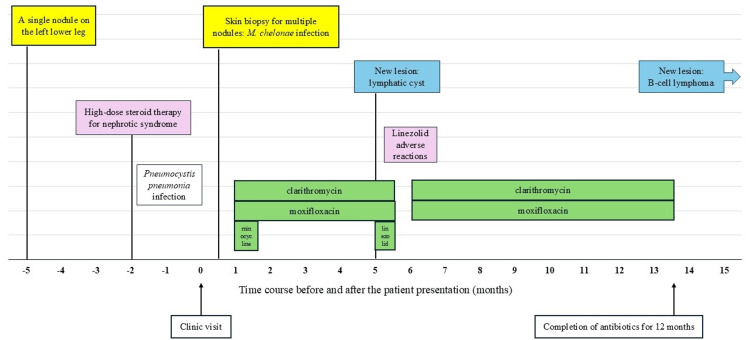
The time course of symptoms, investigations, antibiotic treatments, and outcomes.

The findings were consistent with those of mycobacterial infections. An acid-fast bacillus (AFB) culture was positive after one week. Four weeks after the initial visit, the isolated pathogen was identified as *M. chelonae* using matrix-associated laser desorption/ionization time-of-flight mass spectrometry. The patient was empirically treated with oral clarithromycin, moxifloxacin, and minocycline at doses adjusted for his compromised renal function. *M. chelonae* was later found to be susceptible to clarithromycin, moxifloxacin, and linezolid but resistant to tetracycline (Brosmic RGM®, Kyokuto Pharmaceutical INC, Tokyo, Japan). As the lesions subsided and the patient was doing well, oral minocycline was discontinued (Figure 3).

After four months of treatment with clarithromycin and moxifloxacin, the patient developed a new nodule on his left thigh that gradually enlarged into a palpable mass-like lesion. Owing to the potential risk of *M. chelonae* acquiring drug resistance and the higher risk of the patient contracting infections caused by other pathogens because of his immunosuppressive status, oral linezolid was added to the treatment protocol. Needle aspiration was performed on the new lesion, which yielded a clear serous fluid. The AFB smear was negative, while no bacterial growth was observed in the culture study. Magnetic resonance imaging revealed a cystic lesion consistent with a lymphatic cyst. Following the administration of linezolid, the patient developed adverse reactions, including severe diarrhea, nausea, cytopenia, and a black hairy tongue within two weeks. The patient could not tolerate oral intake; thus, treatment with all three antibiotics was temporarily discontinued. The general condition of the patient improved soon after antibiotic therapy was stopped. As the lymphatic cyst was not of infectious etiology, linezolid was discontinued, whereas moxifloxacin and clarithromycin were resumed for a total of 12 months of therapy. After treatment completion, multiple nodules improved. At the last follow-up, which was three months after the completion of treatment, there was no recurrence of infection. However, the patient developed a new mass lesion on his left thigh, which was confirmed as B-cell lymphoma after the skin biopsy. The patient was referred to another hospital, considering palliative care.

## Discussion

This case demonstrates an *M. chelonae* cutaneous infection in a severely immunocompromised patient that was initially difficult to diagnose owing to its unique presentation. *M. chelonae* infections primarily develop in immunocompromised patients, especially those using systemic steroids [6]. Notably, a recent review study reported that 61% of patients with *M. chelonae* infections were immunocompromised [4]. *M. chelonae* causes cutaneous infections, with disease progression varying depending on the immune status of the host, ranging from chronic nonhealing ulcers [7] to dissemination with fatal outcomes [8]. The progression of *M. chelonae* cutaneous infections is typically chronic, with the period between onset and medical evaluation ranging from months to years [9]. In the present case, only one nodule was detected at the initial visit, which remained unchanged for five months and progressed over two weeks, which was an unusual presentation. In general, an infectious etiology is not suspected if a single nodule is present for an extended period; hence, we initially suspected postinflammatory scarring or a tumor. In fact, several studies have reported that the diagnosis of *M. chelonae* infection was difficult because the presentation was atypical, requiring intensive evaluation and delaying appropriate treatment. In these studies, *M. chelonae* infections initially mimicked other diseases such as vasculitis, pyoderma gangrenosum, varicella, and mycosis [6,8,10-12]. In another case, the diagnosis of cutaneous *M. chelonae* infection was not made initially and topical steroids were empirically administered for an extended period, which might increase susceptibility to *M. chelonae* infection [13]. If clinicians do not suspect *M. chelonae* infection in the differential diagnosis or consider the worsening cutaneous lesions to be attributed to an uncontrolled original disease, increasing the dose of immunosuppressive therapy may lead to the dissemination of *M. chelonae*. In immunocompromised patients, even if the nodule is single and stable for months, clinicians should consider *M. chlonae* infection as a differential diagnosis and perform a skin biopsy with appropriate testing, including AFB staining and culture [11].

No guidelines are currently available for the treatment of NTM skin or soft-tissue infections. In addition, NTM species identification and antibiotic susceptibility testing can take weeks to months, with treatments varying widely for each pathogen. However, in clinical practice, patients need to be initiated on empirical antibiotic therapy before the causative pathogen is identified, as delayed treatment can lead to fatal outcomes or dissemination to other organs, especially in immunocompromised patients. Therefore, accumulating more experience in the treatment of NTM skin or soft-tissue infections, including those caused by *M. chelonae*, is urgently needed.

Treatment options for *M. chelonae* infection include tobramycin, amikacin, imipenem, tigecycline, clarithromycin, linezolid, moxifloxacin, and doxycycline, depending on the susceptibility testing results [14]. Clarithromycin has been a key drug since a clinical trial demonstrated its efficacy against *M. chelonae* cutaneous infection [15]; however, monotherapy is not recommended because of the potential risk of developing drug resistance due to a single point mutation at position 2058 of the 23S rRNA [16]. Currently, the suggested regimen for treating advanced disease consists of the administration of two to three parenteral antibiotics, including macrolides and aminoglycosides, for at least two to six weeks, followed by oral antibiotics for six months [6].

In the present case, based on the susceptibility test results, the patient was treated with oral clarithromycin and moxifloxacin, which led to a successful cure despite the rapidly progressing multiple skin lesions. Previous studies have shown that the same regimen, clarithromycin and moxifloxacin, was effective against focal *M. chelonae* cutaneous infections in immunocompetent patients [17,18]. However, no previous study explored whether this regimen is effective in immunocompromised patients. In previous studies of *M. chelonae* cutaneous infections in immunocompromised patients, patients were treated with multiple antibiotic regimens, including parenteral antibiotics [17,19]. However, the long-term use of multiple antibiotics increases the risk of adverse events, hence leading to antibiotic modification or discontinuation [17]. In a study reporting drug-associated adverse events, both clarithromycin and moxifloxacin administration were associated with extremely low adverse events; the adverse event and permanent drug discontinuation rates were 5.2% and 3.3% for clarithromycin and 3.1% and 2.9% for moxifloxacin, respectively [20]. In contrast, linezolid was associated with a high incidence of adverse events (17.9%) and a permanent discontinuation rate of 14.1% [20]. These results support our hypothesis that oral clarithromycin and moxifloxacin combination therapy is safe and effective for treating rapidly progressive cutaneous *M. chelonae* infections, even in severely immunocompromised patients. Furthermore, this oral regimen can prevent hospitalization when administered as an initial treatment. Clinicians may consider the combination of oral clarithromycin and moxifloxacin as an empirical initial treatment regardless of the immune status of the host when patients present with *M. chelonae* cutaneous infections in areas where clarithromycin and moxifloxacin susceptibility is dominant. To further verify the effectiveness of this regimen, large-scale cohort studies are required.

## Conclusions

In conclusion, *M. chelonae* cutaneous infection rarely presents as a chronically stable single nodule mimicking noninfectious etiologies. Clinicians should be aware of this condition and have a low threshold for performing biopsies, especially in immunocompromised patients, for the early diagnosis and prevention of disease progression. The combination of oral moxifloxacin and clarithromycin is safe and effective against rapidly progressive *M. chelonae* cutaneous infections in immunocompromised patients. This regimen should be considered an empirical treatment option if susceptibility is preserved.
